# Recurrent synapses and circuits in the CA3 region of the hippocampus: an associative network

**DOI:** 10.3389/fncel.2013.00262

**Published:** 2014-01-08

**Authors:** Caroline Le Duigou, Jean Simonnet, Maria T. Teleñczuk, Desdemona Fricker, Richard Miles

**Affiliations:** Centre de Recherche de l’Institut du Cerveau et de la Moelle, INSERM U975, CHU Pitié-Salpêtrière, Université Pierre et Marie CurieParis, France

**Keywords:** CA3, recurrent, synapse, circuit, hippocampus, associative

## Abstract

In the CA3 region of the hippocampus, pyramidal cells excite other pyramidal cells and interneurons. The axons of CA3 pyramidal cells spread throughout most of the region to form an associative network. These connections were first drawn by Cajal and Lorente de No. Their physiological properties were explored to understand epileptiform discharges generated in the region. Synapses between pairs of pyramidal cells involve one or few release sites and are weaker than connections made by mossy fibers on CA3 pyramidal cells. Synapses with interneurons are rather effective, as needed to control unchecked excitation. We examine contributions of recurrent synapses to epileptiform synchrony, to the genesis of sharp waves in the CA3 region and to population oscillations at theta and gamma frequencies. Recurrent connections in CA3, as other associative cortices, have a lower connectivity spread over a larger area than in primary sensory cortices. This sparse, but wide-ranging connectivity serves the functions of an associative network, including acquisition of neuronal representations as activity in groups of CA3 cells and completion involving the recall from partial cues of these ensemble firing patterns.

## RECURRENT EXCITATORY SYNAPSES BETWEEN CA3 CELLS: EMERGENCE

**FIGURE 1:**
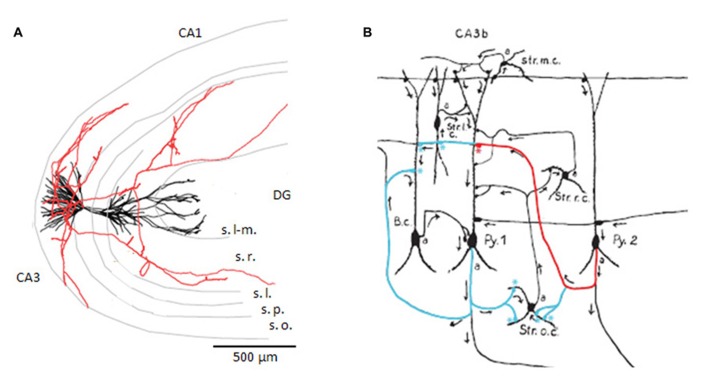
**CA3 pyramidal cell axon and targets.** **(A)** Reconstruction of a CA3 pyramidal cell dendrites, in black, and partial reconstruction of the axon, in red. Adapted from a cell filled by Ishizuka et al. (1995; published as cell c12866 on neuromorpho.org). The CA3, CA1, and dentate gyrus (DG) regions are indicated as are the layers lacunosum-moleculare (s. l-m.), radiatum (s. r.), lucidum (s.l.), pyramidale (s.p.), and oriens (s.o.). **(B)** Drawing of putative axo-dendritic connexions between pyramidal cells (Py. 1 and 2) and interneurons with somata in different layers (B.c., Str. o.c., Str. r.c., Str. l.c., Str. m.c.). The axon of Py. 2 may contact the dendrites of Py. 1, in red, and the interneuron of stratum oriens, in blue. The axon of Py. 1 is drawn contacting the basket cell, in blue (drawing adapted from Lorente de Nó, 1934).

Recurrent connections between CA3 cells in the hippocampus can be seen in early drawings of Golgi stained neurons. Schaffer (1892) and Ramón y Cajal (1899) drew pyramidal cell processes that ramify extensively in the CA3 region as well as projecting into CA1. Later, but still before cellular physiology, Lorente de Nó (1934) drew axonal terminals of a CA3 cell contacting mid-apical dendrites of a nearby pyramidal cell and a basket cell (**Figure 1**). So a basis for recurrent excitation existed before synaptic operations were fully accepted. The absence of this detail did not impede speculation. Recurrent connections between cells of the same region were linked to feedback in chains of connected neurons. Lorente de No (1938) and later Hebb (1949) proposed they might generate reverberating neuronal discharges as an immediate electrical memory.

Intracellular electrophysiology began for the hippocampus with the work of Spencer and Kandel. Initial results dampened the excitation somewhat. They showed that stimulating CA3 cell axons induced dominant inhibitory actions mediated by pyramidal cell excitation of interneurons (Spencer and Kandel, 1961). However recurrent actions were soon linked to reverberation and epileptic synchrony (Kandel and Spencer, 1961). This link was later strengthened by work on epileptiform synchrony induced by penicillin an early antagonist of inhibitory synaptic actions (Lebovitz et al., 1971). Explicitly combining computer simulations and *in vitro* physiology, Traub and Wong (1982) and Wong and Traub (1983) showed how recurrent excitatory synapses might underly delayed all-or-nothing population bursts induced by disinhibition. Physiological support for recurrent synaptic actions came from records of synaptic interactions between CA3 pyramidal cells in slices (Miles and Wong, 1986). Recurrent synapses together with the modeling work could explain the unexpected finding that stimulating a single cell could initiate interictal-like bursts of much larger neuronal populations (Miles and Wong, 1983).

## AXONAL DISTRIBUTIONS OF CA3 PYRAMIDAL CELLS

Axons of single CA3 pyramidal cells of the rat (**Figure 1**) and guinea-pig have been traced from neurons filled with biocytin or horseradish peroxidase (Ishizuka et al., 1990; Sik et al., 1993; Li et al., 1994; Wittner et al., 2006a; Wittner and Miles, 2007). Before projecting out of the region, axons ramify in stratum oriens and radiatum of CA3 contacting apical and basilar dendrites of other pyramidal cells as well as interneurons. Typically they divide into 5–10 collaterals projecting in different directions but rarely returning towards their parent neuron. Longitudinal projections of single axons (Lorente de Nó, 1934) can extend for ~70% of the dorso-ventral extent of rodent hippocampus (Sik et al., 1993; Li et al., 1994). A significant proportion of synapses made by a CA3 pyramidal cell may contact other CA3 cells. The Li et al. (1994) estimated 30–70%. Other connections are made onto CA1 neurons, while there is also a strong commissural projection.

The total axonal length of well-filled CA3 pyramidal cell arbors is estimated as 150–300 mm in the rat with about 30% of the ramification within CA3 (Ishizuka et al., 1990; Li et al., 1994). Terminals are present along all of this distance and a single pyramidal cell is estimated to form 30,000 to 60,000 terminals. Terminals have been thought to target pyramidal cells and interneurons with a frequency similar to the presence of these neuronal types. Recent data suggest some interneuron subtypes may be selectively innervated (Wittner et al., 2006b). Intra-regional differences exist: CA3b pyramidal cells tend to innervate targets in stratum oriens and radiatum about equally, while CA3a pyramidal cell axons target stratum oriens targets more than those in stratum radiatum (Wittner and Miles, 2007).

## CA3 PYRAMIDAL CELL AXON PHYSIOLOGY

Axon collaterals of CA3 pyramidal cells are un-myelinated. They include Schaffer collaterals that project to CA1 as well as those that ramify within the CA3 region. Action potentials are initiated at ~30–40 μm from the soma, where sodium (Na) channel density reaches a peak according to physiology and immunostaining (Meeks and Mennerick, 2007). In regions beyond the action potential initiation site, recurrent axons of CA3 pyramidal cells conduct at velocities of 0.2–0.4 mm/ms (Soleng et al., 2003b; Meeks and Mennerick, 2007).

The Na channels expressed by CA3 recurrent collaterals seem likely to be Nav1.2 and Nav1.6 (Royeck et al., 2008; Debanne et al., 2011). These axons express multiple voltage-gated potassium (K) channels including Kv1.1, Kv1.2, and Kv1.4 (Lorincz and Nusser, 2008), ID (Saviane et al., 2003) the M-channel (Kv7/KCNQ Vervaeke et al., 2006), and the hyperpolarization activated h-current (Soleng et al., 2003a). This diversity of channel expression provides multiple means to modulate action potential shape and so control transmitter release (Bischofberger et al., 2006). Action potential modulation by axonal K-channels may become a total suppression of transmission when an IA-like K-current is fully activated (Debanne et al., 1997; Kopysova and Debanne, 1998).

## CA3 PYRAMIDAL CELL TERMINALS: NUMBERS, FORM, CONTENTS, CHANNELS AND RELEASE

**FIGURE 2:**
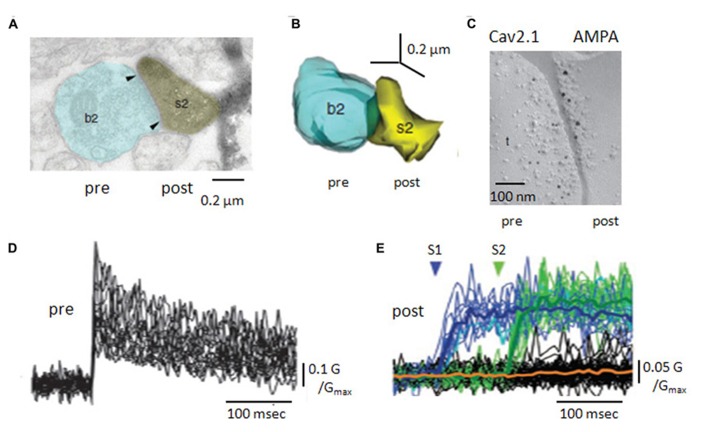
**Anatomy and Ca handling at recurrent synapses between CA3 pyramidal cells.** **(A)** Electron microscopy of a recurrent terminal, b2, apposed to a CA3 pyramidal cell dendritic spine, s2. **(B)** Three-dimensional reconstruction of the contact. The area of the active zone [arrows in **(A)**] was 0.10 μm^2^. **(C)** Double immuno-staining of SDS-digested freeze fracture replica of a recurrent synapse. The smaller gold particles label Cav2.1 molecules (pre) and the larger gold particles recognize a pan-AMPA antibody (post). **(D)** Pre-synaptic Ca transients, measured as changes in fluorescent intensity, for 25 axon terminals of a CA3 pyramidal cell. **(E)** Post-synaptic Ca transients, in response to two pre-synaptic stimuli. Note the occurrence of failures in both post-synaptic responses but their absence from pre-synaptic signals (adapted with permission from Holderith et al., 2012).

Varicosities are formed at distances of 2–5 μm all along CA3 recurrent axons. They often have an ovoid form of diameter ~0.4 μm compared to an axonal diameter of ~0.2 μm (Sik et al., 1993; Li et al., 1994; Wittner and Miles, 2007). Electron microscopy (EM; **Figure 2**) indicates they possess attributes of pre-synaptic boutons with active zones and synaptic vesicles and they face densities at post-synaptic sites (Schikorski and Stevens, 1997; Shepherd and Harris, 1998; Holderith et al., 2012). While varicosities may contain up to three to four active sites, typically they have just one. Synaptic vesicles in recurrent terminals have diameters of 20–40 nm. A terminal may contain up to 800 vesicles with a mean number of 150–270 vesicles.

A small proportion of vesicles are so close (~5 nm) to pre-synaptic membrane that they are considered to be “docked” or available for release. The number of docked vesicles is estimated at 1–15 per terminal (Schikorski and Stevens, 1997; Shepherd and Harris, 1998; Holderith et al., 2012). Vesicles in terminals of CA3 pyramidal cell axons express the transporters, VGLUT1 and 2, and so presumably contain glutamate (Herzog et al., 2006). EM studies on CA3 axon terminals have not revealed a distinct population of large dense-core vesicles, which might contain peptides or other co-transmitters. About half of recurrent terminals contain one mitochondrion (Shepherd and Harris, 1998) and smooth endoplasmic reticulum is typically present: both organelles contribute to calcium (Ca) homeostasis (Sheng and Cai, 2012).

Ca entry into presynaptic terminals triggers transmitter release. CA3 axonal terminals express multiple Ca channel subtypes including Cav2.1, Cav2.2, Cav2.3 (Holderith et al., 2012), as do the mossy fiber terminals that also terminate on CA3 pyramidal cells (Li et al., 2007). Freeze-fracture replica gold immuno-labeling (**Figure 2**) suggests a single terminal expresses several tens of Cav2.1 channels (Holderith et al., 2012). This is more, but not many more, than estimates of the number of Ca-channels needed to trigger release from hippocampal inhibitory terminals (Bucurenciu et al., 2010). Possibly, an elevated Na channel density in terminals enhances Ca entry by boosting depolarization due to axonal spikes (Engel and Jonas, 2005). Certainly, recurrent terminals express various types of K channel which control transmitter release by limiting terminal depolarization. They may include the delayed rectifier type channels Kv1.1 and Kv1.2, the fast-inactivating A-type channel Kv1.4 (Debanne et al., 1997; Kopysova and Debanne, 1998; Lorincz and Nusser, 2008; Palani et al., 2010) as well as K-channels sensitive to both Ca and voltage (Saviane et al., 2003; Raffaelli et al., 2004) and the muscarine sensitive M-channel Kv7/KCNQ (Vervaeke et al., 2006).

Ca changes induced in local recurrent terminals by pyramidal cell firing have been resolved by imaging (Holderith et al., 2012; Sasaki et al., 2012). A single action potential induces a Ca signal of rise time less than 1 ms that decays over several 10 s of ms (**Figure 2**). Ca entry occurs without failure even if it varies between trials at the same terminal and Ca elevations at neighboring terminals are poorly correlated. For a given terminal, the mean amplitude of Ca-signals is better correlated with the area of the active zone than terminal volume (Holderith et al., 2012).

CA3 axon terminals express receptors for transmitters which modulate Ca entry or later steps in release processes (**Figure 2**). Receptors for the metabotropic glutamate receptor, mGluR7, expressed at active zones facing interneurons but not principal cells (Shigemoto et al., 1996) specifically control the excitation of inhibitory cells (Scanziani et al., 1998). The kainate receptor GluK1, reduces release by effects on both Ca entry and on G-protein mediated stages in transmitter release (Salmen et al., 2012). In contrast, presynaptic NMDA receptors enhance Ca entry and facilitate release at some synapses made by CA3 collaterals(McGuinness L et al., 2010).

## PRE- MEETS POST: SYNAPSES MADE BY CA3 PYRAMIDAL CELLS WITH OTHER CA3 CELLS

When a single spike induces Ca entry into a CA3 axon terminal, one, or none, or several vesicles of the excitatory transmitter glutamate are liberated. Release fails, when Ca enters a terminal but no transmitter is liberated, as shown by imaging Ca-entry (**Figure 2) via post-synaptic glutamate receptors (Koester and Johnston, 2005**; Holderith et al., 2012). Multi-vesicular release following a single action potential is most convincingly demonstrated when two distinct post-synaptic events can be resolved in time, as at some inhibitory synapses in the cerebellum (Auger et al., 1998). Analysis of variations in synaptic events over a range of liberation probabilities supports multi-vesicular liberation (Conti and Lisman, 2003; Christie and Jahr, 2006).

Glutamate, released from a pre-synaptic terminal, binds to post-synaptic receptors. The number of receptors per site has been estimated with physiological, imaging, and anatomical techniques. Post-synaptic sites facing terminals of CA3 pyramidal cell axons in young animals, all express NMDA (*N*-methyl-D-aspartate) receptors (Takumi et al., 1999). Glutamate uncaging onto post-synaptic sites activates 3–10 NMDA receptors (Nimchinsky et al., 2004). Semi-quantitative immunostaining studies and imaging agree that about 30% of post-synaptic sites possess no AMPA (α-amino-3-hydroxy-5-methyl-4-isoxazolepropionic acid) receptors (Nusser et al., 1998; Takumi et al., 1999; Nimchinsky et al., 2004). At synapses where AMPA receptors are expressed, about 10 of them (**Figure 2**) are estimated to be activated after a single pre-synaptic spike in acute slices (Nimchinsky et al., 2004), 40–150 in culture (Matsuzaki et al., 2001). AMPA receptors are present at recurrent synapses with most types of interneuron (Nusser et al., 1998). NMDA receptors are less frequently expressed at synapses with interneurons and may be absent at contacts with fast-spiking, parvalbumin containing cells (Nyiri et al., 2003).

There are two other important differences between synapses made with interneurons and with pyramidal cells. First, recurrent contacts tend to innervate pyramidal cell spines, while those with most types of inhibitory cell innervate dendritic shafts (Gulyas et al., 1993; Freund and Buzsáki, 1996). Second, the AMPA receptor isoforms involved are different. AMPA receptor complexes at synapses formed with interneurons do not include the GluR2 subunit (Bochet et al., 1994; Geiger et al., 1995), resulting in faster kinetics (Miles, 1990), Ca-permeability, and a block by endogenous intraneuronal polyamines (Isaac, 2007).

## PRE- MEETS POST IN DUAL RECORDS

Double records from pre- and post-synaptic neurones at recurrent synapses between CA3 cells were first made to prove their existence directly. They remain the most persuasive means to examine how one neuron influences another. They have permitted definition of the number of synaptic contacts involved in a unitary connection and assessment of variability and changes in synaptic efficacy (Debanne et al., 2008).

**FIGURE 3:**
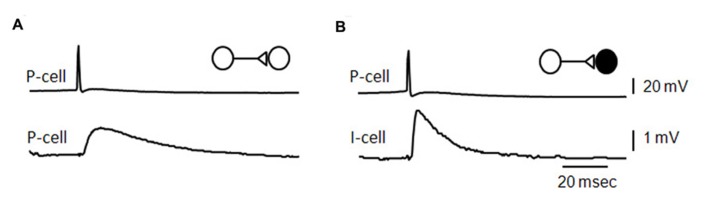
**Unitary effects of recurrent excitatory synapses.** **(A)** Average of EPSPs initiated in a CA3 pyramidal cell by single action potentials in a pre-synaptic pyramidal cell **(B)**, average of EPSPs elicited in a fast-spiking CA3 interneuron by action potentials in a pyramidal cell [an unpublished data Miles and Wong **(B)** adapted from Miles, 1990].

Records from pairs of CA3 pyramidal cells in acute slices (**Figure 3**) suggest one pyramidal cell excites 2–3% of possible pyramidal cell targets in a slice (Miles and Wong, 1986; Miles and Wong, 1987b). Odds are more favorable in organotypic slices. Connectivities are 30–60% (Debanne et al., 1995; Pavlidis and Madison, 1999). The number of release sites involved in a connection may also be higher in organotypic cultures. One to three contacts have been validated by EM for synapses between pyramidal cells and interneurons recorded and filled with biocytin in slices. In contrast, light microscopy suggests 14–19 putative contacts may be involved in connections between CA3 pyramidal cells in organotypic culture (Pavlidis and Madison, 1999).

The mean amplitude of synaptic potentials is about 1 mV at connections between pyramidal cells in acute slices (Miles and Wong, 1986) and in culture (Debanne et al., 1995). EPSPs induced in fast-spiking interneurons (**Figure 3**) are larger and faster than those initiated in pyramidal cells. Unitary synaptic current amplitude at connections made in culture can vary in the range 10–200 pA with an average near 30 pA (Pavlidis and Madison, 1999; Sasaki et al., 2012). In records from both acute slices and culture, events initiated successively at the same connection vary in amplitude. Transmission can fail, more often at connections with smaller averaged events. However pre-synaptic Ca entry never fails, even though it varies between successive action potentials (Holderith et al., 2012; Sasaki et al., 2012) and Ca signals are higher at terminals with a higher release probability (Koester and Johnston, 2005).

Synaptic events initiated sequentially at the same site vary in amplitude. This variability may have both pre- and post-synaptic components (Silver et al., 2003; Biró etal., 2005). Clear data on post-synaptic variability, is facilitated at connections with a single identified release site. At such a synapse, the variability in size of post-synaptic events was estimated at 20–50% (Gulyas et al., 1993). This variability might emerge from differences in the number of transmitter molecules released or in the activation of post-synaptic receptors.

The properties of recurrent synapses differ quite markedly from those of mossy fiber inputs, the other major source of excitation of CA3 pyramidal cells. A mossy fiber may make 10–20 connections with different CA3 pyramidal cells (Claiborne et al., 1986). A recurrent collateral makes several thousand contacts with a much larger target population. Mossy fiber boutons contact proximal apical dendrites of CA3 pyramidal cells and have a diameter of 4–8 μm. Each bouton may include 20–30 active zones, whereas a recurrent synapse may make one to three terminals on a post-synaptic cell. Finally mossy fibers contact apical dendrites near the CA3 soma, while recurrent synapses terminate at more distant dendritic sites resulting in smaller, slower somatic synaptic events. A mossy fiber input from one dentate granule cell can induce CA3 pyramidal cell firing and can so be termed a “detonator” synapse (Henze et al., 2002), whereas multiple spikes are needed to induce firing at recurrent synapses (Miles and Wong, 1987a).

## SHORT-TERM AND LONG-TERM SYNAPTIC PLASTICITY IN DOUBLE RECORDINGS

Records from pre- and post-synaptic cells at recurrent synapses have offered novel insights into activity dependent changes in synaptic strength over times lasting from milliseconds to hours.

Short-term plasticity (milliseconds to seconds) results from at least two functionally opposing processes. First, a single spike may facilitate transmission when the same synapse is activated again (Ddel Castillo and Katz, 1954). An enhanced release probability over several tens of milliseconds is ascribed to a residual elevation of intra-terminal Ca (Holderith et al., 2012; Sasaki et al., 2012). Second, and inversely, depression may result if few vesicles are available for release (Schikorski and Stevens, 1997; Shepherd and Harris, 1998). If they are replaced slowly (Stevens and Tsujimoto, 1995; Staley et al., 1998) the probability of a second release may be reduced by depletion. Both processes occur at connections between CA3 pyramidal cells (Debanne et al., 1996; Pavlidis and Madison, 1999). When a first spike induces a large event, a second synaptic response tends to be smaller due to depletion. Inversely a second EPSP tends to be larger after a small first event due to the residual Ca enhancement of release probability. Reflecting the underlying mechanisms, facilitation is maximal at 20–70 ms and terminates at about 500 ms, while depression can take several seconds to recover completely.

Long-term plasticity (minutes to hours) at different synapses varies in mechanisms of induction and expression. One of the most studied forms, long-term synaptic potentiation at Schaffer collateral synapses made by CA3 pyramidal cells with CA1 cells, is induced via the activation of NMDA receptors and expressed as the post-synaptic recruitment of AMPA receptors (Kerchner and Nicoll, 2008). Long-term changes in synaptic efficacy seem to depend on similar mechanisms at recurrent synapses between CA3 pyramidal cells. Paired records from coupled CA3 cells have revealed some unitary details of this synaptic plasticity. The same connection can be potentiated or depotentiated (Debanne et al., 1998) by different temporal patterns of paired pre- and post-synaptic firing. About 20% of unitary interactions depend exclusively on NMDA receptors before potentiation (Montgomery et al., 2001), while both AMPA and NMDA receptors are activated after potentiation. Weak connections potentiate to a larger degree than initially strong connections (Debanne et al., 1999; Montgomery et al., 2001). Finally some connections between CA3 pyramidal cells do not seem to potentiate at all (Debanne et al., 1999; Montgomery and Madison, 2002).

## TRANSMISSION OF RECURRENT EXCITATORY SIGNALS ON THE MEMBRANE OF A POST-SYNAPTIC CELL

Activation of membrane currents intrinsic to a post-synaptic cell by recurrent EPSPs affects how they sum, spread and eventually initiate firing. Initial evidence came from a prolongation of the decay of unitary EPSPs induced by pyramidal cell depolarization at subthreshold membrane potentials (Miles and Wong, 1986). In contrast unitary EPSPs initiated in fast-spiking inhibitory cells were not prolonged at depolarised subthreshold potentials (Miles, 1990). Work combining somatic records and synaptic stimuli with cell-attached records from dendrites, showed the activation of both inward currents, probably persistent Na channels, low-threshold Ca channels (Magee and Johnston, 1995), and outward currents, both inactivating and persistent (Hoffman et al., 1997). These currents have been more precisely described for EPSPs initiated by Schaffer collaterals (Lipowsky et al., 1996; Andreasen and Lambert, 1999; Perez-Rosello et al., 2011), as has evidence for a dendritic expression of the *I*–*h* current (Magee, 1999).

Distinct currents have been associated with specific effects on EPSP shape, summation, and spread. Na-channel activation near the peak of an EPSP tends to increase amplitude, while Ca-channels activated during the decay phase act to prolong EPSPs. The striking increase in dendritic expression of the *I*–*h* channel with distance from the soma (Lörincz et al., 2002) tends to equalize EPSPs impinging at proximal and distal sites (Magee, 1999). Dendritically expressed inactivating K-channels have been linked to less-than-linear summation of paired EPSPs impinging on different dendrites (Urban and Barrionuevo, 1998). Dual records from the soma and apical dendrites of CA3 pyramidal cells disclose two distinct regions of dendritic excitability (Kim et al., 2012). Fast Na-spikes are more easily initiated at distant sites corresponding to zones of recurrent synaptic inputs, while excitability of more proximal dendritic sites is lower.

The role of intrinsic currents in shaping interneuron EPSPs may be quite different to that in pyramidal cells. Simulated EPSPs induce purely inward currents in pyramidal cells but rather induce inward-outward current sequences in interneurons (Fricker and Miles, 2000). So, while, EPSPs in pyramidal cells are prolonged, EPSPs in interneurons may decay more rapidly due to the activation of an outward current at subthreshold potentials.

Synaptic inputs to a neuron are significant to surrounding cells when they initiate firing. Summed EPSPs initiated by repetitive firing of a single CA3 pyramidal cell sometimes induce cause a post-synaptic pyramidal cell to fire (Miles and Wong, 1986). Spike-to-spike latencies are 10–15 ms, consistent with a role for recurrent excitatory synapses in the genesis of delayed (50–100 ms) population bursts (Traub and Wong, 1982; de la Prida et al., 2006). Recent work suggests spike-to-spike transmission may be limited to a few strong connections (Ikegaya et al., 2013).

**FIGURE 4:**
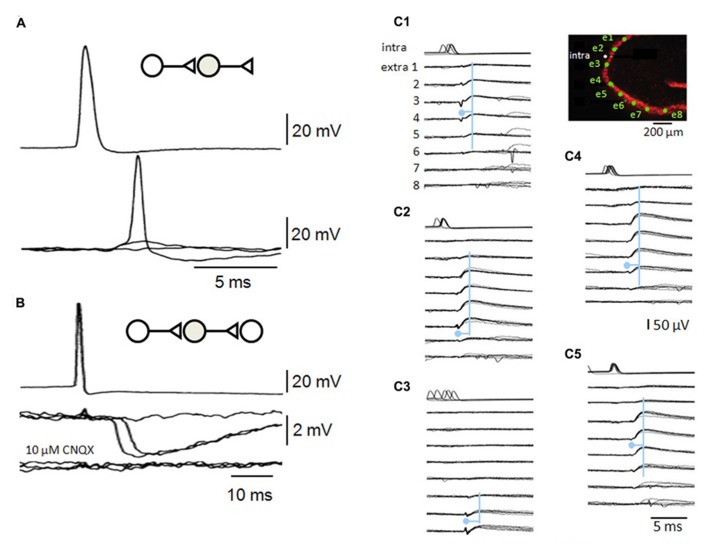
**Recurrent inhibitory circuits in the CA3 region.** **(A)** Post-synaptic responses of a fast-spiking interneuron to single pre-synaptic action potentials in a CA3 pyramidal cell. Responses include a failure of transmission, an EPSP and an EPSP that initiates interneuron firing. **(B)** Di-synaptic inhibitory interactions between two CA3 pyramidal cells. Single action potentials in one cell induce IPSPs at variable latencies consistent with that of firing in **(A)**, as well as some failures. Di-synaptic IPSPs were suppressed by the glutamate receptor blocker CNQX. **(C)** A single pyramidal cell can initiate multiple di-synaptic IPSPs via firing in distinct interneurons. Records from a pyramidal cell (intra) and extracellular records from eight sites in st. pyramidale (extra 1–8, the diagram shows st. pyramidale in red and electrode sites in green). Field IPSPs were detected on electrodes 1–6 **(C1)**, 2–7 **(C2)**, 6–8 **(C3)**, 1–7 **(C4)**, and 2–6 **(C5)** repeatably following single action potentials (traces are aligned on six overlapping field IPSPs for each trace). Field IPSPs are preceded by extracellular action potentials of short duration on electrodes 2–3 **(C1)**, 6 **(C2)**, 7–8 **(C3)**, 6–7 **(C4)**, and 5–6 **(C5)**. The pyramidal cell may have initiated five distinct di-synaptic inhibitory interactions in these slice records (see Bazelot et al., 2010).

Pyramidal cells induce interneuron firing more effectively and at shorter latencies of 1–3 ms (Miles, 1990; Csicsvari et al., 1998; Cohen and Miles, 2000). Interneuron EPSPs are larger and faster than recurrent EPSPs in pyramidal cells, and interneuron firing threshold is lower (**Figure 4**). When interneurons are excited to fire, pyramidal cells may trigger di-synaptic IPSPs (**Figure 4**) with high probability and considerable divergence (Miles, 1990; Csicsvari et al., 1998; Bazelot et al., 2010). While EPSP boosting mechanisms in interneuron dendrites are not clear, it is surprising that EPSPs induced from a single site (Gulyas et al., 1993) can induce firing. Even so, EPSP-spike coupling at single release site excitatory synapses with some cerebellar interneurons (Carter and Regehr, 2002) is also sufficiently strong that EPSPs control the timing of interneuron firing.

## RECURRENT EXCITATORY CONTRIBUTIONS TO POPULATION ACTIVITIES IN THE CA3 REGION

Recurrent synapses transmit excitation from CA3 pyramidal cells to other pyramidal cells and to interneurons. They play a key role in operations and functions of the CA3 region, including the generation of physiological and pathological synchronous population activities.

## INTERICTAL EPILEPTIFORM RHYTHM

A key finding linking recurrent excitation to epileptiform activity was that stimulating any afferent pathway induced epileptiform firing in CA3 (Ayala et al., 1973). Population bursts occurred with a variable delay of 20–100 ms after the afferent response. Traub and Wong (1982) suggested that during the delay recurrent synaptic interactions within the CA3 population generate a population synchrony. Synchrony induced in disinhibited slices is complete in that all neurons tend to fire together with a field potential decorated with high frequency oscillations (Jefferys et al., 2012). Traub and Wong suggested recurrent circuits should possess two properties to generate such an event. Recurrent contacts should be divergent and one cell could cause more than one target neuron to fire. These points were verified with the demonstration that some single pyramidal cells could induce or entrain inter-ictal-like events (Miles and Wong, 1983, 1986, 1987a; de la Prida et al., 2006). Di-synaptic feedback inhibition via CA3 pyramidal cell excitation of feedback interneurons, was shown to prevent the spread of firing by recurrent excitatory pathways (Miles and Wong, 1986, 1987a,b).

Recurrent synaptic function controls several features of the epileptiform activity induced by disinhibition. The duration of the population burst (20–80 ms) has been shown to result from transmitter depletion (Staley et al., 1998). The delay from one burst to the next (1–10 s) depends on the time for docked vesicles to be replenished (Staley et al., 1998; Staley et al., 2001). Procedures that induce persistent synaptic changes have persistent effects on the strength and frequency of network burst firing (Bains et al., 1999; Behrens et al., 2005).

Cellular properties also affect disinhibition induced synchrony by controlling transmission in chains of connected neurons. In slices, population bursts tend to be initiated in the CA3a region, where cellular excitability and recurrent connectivity are high (Wittner and Miles, 2007). In CA3a, spontaneous events are preceded by a field potential of duration about 50 ms (Wittner and Miles, 2007) during which excitatory synaptic events occur with increasing frequency. This delay is similar to that between single cell firing and a population event (Miles and Wong, 1983; de la Prida et al., 2006). Modeling work suggested that during this time activity in the pyramidal cell population increases in non-linear fashion (Traub and Wong, 1982). An epileptiform burst occurs when population activity exceeds a threshold frequency (de la Prida et al., 2006).

## SHARP-WAVE RHYTHM

Sharp waves (O’Keefe and Nadel, 1978; Buzsáki et al., 1992) are field potentials of duration 100–150 ms, corresponding to a partial neuronal synchrony during behaviors including immobility and slow wave sleep. They are initiated in the CA3 region (Csicsvari et al., 2000) and have been associated with the consolidation of acquired events (Girardeau et al., 2009; Jadhav et al., 2012) represented as firing in specific groups of neurons.

Both recurrent excitatory interactions and the actions of specific interneurons have been implicated in the genesis of sharp waves (Buzsáki et al., 1992; Csicsvari et al., 2000). Sharp wave fields are enhanced by inducing long-term changes at recurrent synapses (Behrens et al., 2005). And yet, sharp waves are not identical with epileptiform events and do not depend on recurrent excitation alone (Liotta et al., 2011). Repetitive firing of peri-somatic interneurons may be a crucial element in sharp wave generation (Buzsáki et al., 1992; Klausberger et al., 2003). Gap-junctions have also been associated with sharp-waves, with the observation of “spikelets” in pyramidal cells and a blockade by gap-junction antagonists (Draguhn et al., 1998). However sharp waves persist, at reduced strength, in animals where the gap junction protein connexin 36 is genetically deleted (Pais et al., 2003). Possibly then, recurrent excitation of both pyramidal cells and interneurons (Hájos et al., 2013) may suffice to generate sharp waves.

## THETA AND GAMMA RHYTHMS

In contrast to sharp waves, theta fields (4–12 Hz) are generated when spatial memory representations are first acquired during movements (Vanderwolf, 1969; O’Keefe and Nadel, 1978). Place-cells fire with theta oscillations and theta waves are also detected in rapid eye movement sleep.

Theta oscillations probably depend on signals generated outside the CA3 region. Signals from the septum may provide a sustained cholinergic excitation as well as glutamatergic (Huh et al., 2010) and inhibitory signals which selectively targeting hippocampal interneurons to disinhibit pyramidal cells (Freund and Antal, 1988; Tóth et al., 1997; King et al., 1998). Synaptic connections within the CA3 region probably reinforce the rhythm via reciprocal interactions between pyramidal cells and some, probably peri-somatic, interneurons (Soltesz and Deschênes, 1993).

Gamma oscillations at 30–70 Hz may be superimposed on theta rhythmicity (Bragin et al., 1995; Csicsvari et al., 2003; Hasselmo, 2005). They are suggested to bind, or coordinate, activity of spatially dispersed neurons due to a single stimulus (Gray et al., 1989). In contrast to theta, gamma oscillations are generated within the CA3 region. Reciprocal synaptic interactions between peri-somatic inhibitory cells and CA3 pyramidal cells via recurrent synapses are suggested to contribute both *in vivo* (Csicsvari et al., 2003) and in slice models of gamma induced by cholinergic agonists (Oren et al., 2006) or kainate (Fisahn, 2005). Gap junctions that transmit excitation between CA3 pyramidal cell axons may be another crucial factor in gamma generation (Traub and Bibbig, 2000; Traub et al., 2003).

## COMPARISON OF RECURRENT CONNECTIVITY IN CA3 AND OTHER CORTICAL REGIONS

The hippocampal treatment of events, memories or representations may depend in part on the associative nature of the recurrent excitatory network between CA3 pyramidal cells. How do recurrent circuits in CA3 compare to those in other associative or sensory cortical regions?

The spatial extent of excitatory terminals seems to differ for recurrent synapses in associative, allocortical regions, such as CA3 and the olfactory cortex, and in six-layered primary sensory neocortex. CA3 pyramidal cell axons project longitudinally through most of the hippocampus (Lorente de Nó, 1934; Li et al., 1994). Local axons diffusely cover most of the olfactory cortex (Haberly, 2001; Franks et al., 2011; Poo and Isaacson, 2011). Connectivity within a six-layered cortex is certainly more complex, but overall may be more restrained in space. For instance, axons of layer IV pyramidal cells from sensory cortices tend to ramify locally within modules such as a single somatosensory barrel (Petersen and Sakmann, 2000; Feldmeyer, 2012). Superficial or deep layer pyramidal cells of primary visual or somatosensory cortex make longer range but often patchy projections terminating in regions occupied by cell groups of similar function (Gilbert and Wiesel, 1989; Holmgren et al., 2003; Ko et al., 2011; cf Feldmeyer, 2012)

The density of excitatory connections between pyramidal cells may be somewhat higher in sensory cortical modules than in associative allocortex such as CA3 or piriform cortex. Paired records from acute slices gave a value of 0.02–0.03 for the probability of a connection between two CA3 pyramidal cells (Miles and Wong, 1986) and recurrent connectivity in piriform cortex is estimated at 0.002–0.01 (Franks et al., 2011; Hagiwara et al., 2012). Estimates of connectivity are somewhat higher from paired records in slices of sensory cortex. The probability of connection between cells in different cortical layers ranges from 0.1 to 0.3 (0.2–0.3 in layer 4 of barrel cortex, Lefort et al., 2009; Feldmeyer, 2012; 0.1 in layer 2/3 of neocortex, Holmgren et al., 2003; 0.1 in layer 5 neocortex, Markram et al., 1997).

An alternative way to define connectivity could be to measure the spatial distribution of terminals formed by the axon of a single cell. Terminals of some pyramidal cells in sensory cortex (Petersen and Sakmann, 2000) seem likely to show a more focal topology than those of the CA3 region (Ishizuka et al., 1990; Li et al., 1994). Data from paired records in slices indicates a lower local connectivity in CA3 than in sensory cortex. Lower values for recurrent connectivity may be a design feature to ensure sparse representations in an associative region.

Recurrent excitatory synapses may contact cortical interneurons selectively in both associative and sensory cortices. Paired records suggest connectivity from pyramidal cells to fast-spiking interneurons is higher than onto pyramidal cells (0.5–0.7 in neocortex layer 2, Holmgren et al., 2003; in barrel cortex layer 2 ~0.6, Avermann et al., 2012; 0.2 in piriform cortex layer 3, Stokes and Isaacson, 2010). A higher connectivity as well as stronger signaling at single connections with GABAergic interneurons (Helmstaedter et al., 2008) protects against excessive synchrony, maintains stable population firing and sharpens signaling by imposing a sparse coding.

The strength of afferent and recurrent synapses may differ in both associative and sensory cortices. Mossy fiber synapses with CA3 pyramidal cells have more release sites (Claiborne et al., 1986) and stronger actions (Henze et al., 2002). Synapses from olfactory bulb onto piriform cortex cells are both stronger and less numerous that recurrent synapses (Franks et al., 2011; Poo and Isaacson, 2011). In barrel cortex however, recurrent connections between layer 4 pyramidal cells seem to be stronger (Feldmeyer et al., 1999; Feldmeyer, 2012) than thalamic synapses which excite the same cells (Bruno and Sakmann, 2006).

Thus recurrent networks of associative cortical regions have a wider spatial extent and a lower probability of connection between pyramidal cells than those in sensory cortices.

## THE CA3 RECURRENT SYSTEM AS AN ASSOCIATIVE NETWORK

**FIGURE 5:**
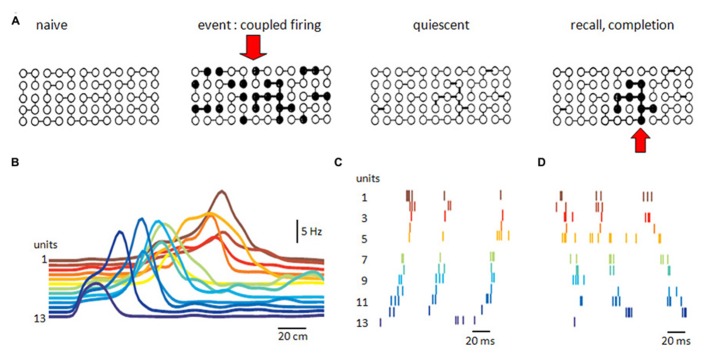
**Recurrent excitatory networks.** **(A)** Possible schema of connectivity and operations in a recurrent neuronal network. Some neurons are connected in the naïve network. Coupled firing in a subset of neurons during an event reinforces synapses between them. Reinforcement persists during quiescence, until partial activation recalls or completes firing of the neuronal subset associated with the original event. **(B)** Sequential firing of 13 pyramidal place cells as an animal passes through a space (horizontal axis is distance). Reactivation of sequential firing of these cells as **(C)** forward replay or **(D)** backward replay (adapted with permission from Diba and Buzsáki, 2007).

Associative synaptic networks have been linked to the processes of completion and recall of stored information (**Figure 5**). McNaughton and Morris (1987) noted that similar hypotheses have often been discovered. What do they assume? And how might they be tested?

Such hypotheses suppose that information, or a representation, or an event, or a memory, has a distributed existence as the correlated, or synchronous, discharge of a group of neurons (Hebb, 1949; Marr, 1971). Different informations presumably involve different groups, raising the question of how representations are constrained to be neuronally orthogonal (Marr, 1971; Rolls and Treves, 1998). They suppose that a way exists to associate or strengthen synaptic relations within such a group or ensemble of synchronously active neurons. It might correspond to the persistent synaptic potentiation which occurs when pre- and post-synaptic cells fire together (Hebb, 1949; Bliss and Lomo, 1973). They suppose that a full representation of an event can be recalled from some of its elements (Gardner-Medwin, 1976; McNaughton and Morris, 1987). The CA3 recurrent network where activity in some single cells can trigger population activities (Miles and Wong, 1983; Fujisawa et al., 2006) might be capable of operations similar to a cued recall (**Figure 5**). The spatially widespread but lower connectivity of associative recurrent networks may favor this form of information storage.

Improved techniques to record and manipulate activity in large groups of neurons begin to suggest distributed ensembles may contribute to storage and recall. Using tetrodes to separate firing in 50–100 single units, Wilson and McNaughton (1994) showed that CA1 place-sensitive neurons that fired together during a spatial behavior, discharged synchronously again during the following episode of sleep. Correlated firing in cell pairs was increased as animals learned a task and maintained during replay. A specific role for recurrent synapses was established by genetically deleting NMDA receptor expression at recurrent synapses of CA3 pyramidal cells (Nakazawa et al., 2002, 2003). With the basis for persistent changes abolished, recall of spatial memories from partial cues was suppressed. Optical stimulation has recently been used to re-activate neurons associated with a representation (Liu et al., 2012). An ensemble of granule cells active during fear conditioning was labeled with a construction including c-fos which also induced expression of a light-sensitive opsin. Re-activating the sparse granule cell ensemble optically later, induced a fear response in a different context.

These data point to distinct neuronal operations associated with acquisition and recall. A two-stage memory system has often been postulated (James, 1890; Buzsáki, 1989). The two stages may occur during distinct brain and behavioral states. External representations, especially those associated with space (O’Keefe and Nadel, 1978) and possibly also time (Huxter et al., 2003; Kraus et al., 2013) are acquired during theta activity. In contrast, recall or consolidation is linked with sharp-waves generated in CA3 (Buzsáki, 1989). Switching between these opposing behaviors might be achieved with distinct modulatory transmitters (Hasselmo et al., 1995) or, perhaps more economically, by external control of specific interneurons (Viney et al., 2013).

Acquisition and replay of ensemble activity were first described during theta and sharp waves respectively (Wilson and McNaughton, 1994). Several variants of the exact replay of neuronal firing sequences have now been distinguished most often in CA1 during sleep (Lee and Wilson, 2002; cf Matsumoto et al., 2013) and the awake state (Foster and Wilson, 2006; Diba and Buzsáki, 2007). Firing replay during sharp waves is increasingly linked to the consolidation of a memory or representation by transfer from the hippocampus to a more permanent storage in cortex (Rasch and Born, 2007; Nakashiba et al., 2009; O’Neill et al., 2010). During sharp waves of slow-wave sleep, similar firing sequences are detected in hippocampus and cortex (Ji and Wilson, 2007) and suppressing sharp waves during sleep interferes with consolidation (Girardeau et al., 2009).

The data on these forms of replay raises questions for future work. It needs to be re-examined in CA3. Many, but not all (Diba and Buzsáki, 2007), papers report data from CA1 with the caveat that the activity is likely to have originated in CA3. How is the apparent precision in firing maintained during the translation from CA3 to CA1? How is an appropriate sequence initiated in CA3? What neuronal and synaptic mechanisms can explain how a specific sharp wave is chosen, define the inhibitory and pyramidal cells that fire during it, and permit reversal of this sequence? Better techniques to define cellular and synaptic physiology in context of data on the activity of large numbers of neurons (Matsumoto et al., 2013) will be needed for the next steps to uncover the role of recurrent synapses and the functions of the CA3 region.

## Conflict of Interest Statement

The authors declare that the research was conducted in the absence of any commercial or financial relationships that could be construed as a potential conflict of interest.
