# Erratum: Recurrent synapses and circuits in the CA3 region of the hippocampus: an associative network

**DOI:** 10.3389/fncel.2014.00048

**Published:** 2014-02-20

**Authors:** Caroline Le Duigou, Jean Simonnet, Maria T. Teleńczuk, Desdemona Fricker, Richard Miles

**Affiliations:** Centre de Recherche de l'Institut du Cerveau et de la Moelle, INSERM U975, CHU Pitié-Salpêtrière, Université Pierre et Marie CurieParis, France

**Keywords:** erratum

1. We should like to correct the name for one of the authors. Maria T. Teleñczuk should be corrected to Maria T. Teleńczuk.
2. ACKNOWLEDGMENTS: Maria T. Teleńczuk would also like to acknowledge support from the Ecole des Neurosciences and the DIM Cerveau et Pensée (Paris, Ile de France).

